# Complicated Relationships between Anterior and Condylar Guidance and Their Clinical Implications—Comparison by Cone Beam Computed Tomography and Electronic Axiography—An Observational Cohort Cross-Sectional Study

**DOI:** 10.3390/life13020335

**Published:** 2023-01-26

**Authors:** Łukasz Lassmann, Zuzanna Nowak, Jean-Daniel Orthlieb, Agata Żółtowska

**Affiliations:** 1 Dental Sense Medicover, ul. Myśliwska 33a, 80-283 Gdańsk, Poland; 2 Department of Temporomandibular Disorders, Medical University of Silesia in Katowice, Traugutta sq. 2, 41-800 Zabrze, Poland; 3 Faculty of Odontology, Aix-Marseille University, 58 Boulevard Charles Livon, 13007 Marseille, France; 4 Department of Conservative Dentistry, Faculty of Medicine, Medical University of Gdańsk, 80-210 Gdańsk, Poland

**Keywords:** temporomandibular joint, temporomandibular disorders, dental occlusion, dental prosthesis

## Abstract

A complex prosthodontic treatment is believed to be more successful when the condylar path is replicated using the articulator. However, there is an ongoing major disagreement between the researchers as the exact relationship between the posterior and anterior determinants has not been clear. The purpose of this study was to investigate whether the protrusive movement of the mandible does correlate with the temporomandibular joint (TMJ) anatomy or with incised features. Subjects (15 males and 15 females) were qualified for this study based on an initial interview including the following criteria: age 21–23 (+/−1), no history of trauma, orthodontic treatment, or temporomandibular disorders (TMD). For each patient, the angle of the condylar path, incisal guidance angle (IGA), interincisal angle, as well as overbite and overjet were measured on cone beam computed tomography (CBCT). This was followed by the examination with the Modjaw^®^ electronic axiograph recording and calculating the functional sagittal condylar guidance angle (SCGA) for the right and left TMJ during the protrusion. The results show that the mean functional axiographic measurement of SCGA in protrusion significantly correlates with the TMJ anatomy presented on CBCT. Moreover, a significant correlation was found between the values of SCGA in the functional and anatomical measurements in all its variants. It turned out that, statistically, the AB measurement was the most accurate. Finally, results showed that incisal relationships of permanent teeth such as overbite, overjet, incisal guidance angle and interincisal angle do not correlate with TMJ anatomy, and therefore, regarding an analyzed study group, do not affect the TMJ formation in young adults.

## 1. Introduction

The temporomandibular joint (TMJ) is a part of the stomatognathic system that allows the mandible to move. In adults, those movements are dictated by the shape of the articular tubercle, articular disk, the limitation of the associated ligaments, the neuromuscular system, and the guiding planes of the teeth [1]. A sagittal trajectory of the condyle traversing the articular tubercle with any chosen horizontal plane (e.g., Frankfort) creates the sagittal condylar guidance angle (SCGA). The SCGA can be measured radiographically, using a jaw movement recording device, or with a protrusive interocclusal bite registration method [2,3]. In previous decades, pantomograms have sometimes been recommended for measuring SCG [4]. However, they have many disadvantages, including the orientation of the reference plane and the head, and parallax distortions due to the difficulty of distinguishing the outline of the articular elevation from the lower border of the zygomatic arch [5]. Moreover, panoramic images are often not precise for SCGA measurement due to the overlapping of many structures. In contrast, CBCT scans provide a 3D image for both sides without overlapping, so that articular sublimation and acetabular fossa can be clearly distinguished from adjacent structures, and the CBCT SCGA measurement alone gives much more reliable results. With the advent of CBCT, CT scans were associated with less radiation exposure and greater accuracy, which resulted in their widespread use in dentistry [6].

The registered values of SCGA allow for an accurate articulator setting [3,7,8]. A complex prosthodontic treatment is believed to be more successful when the condylar path is replicated using the articulator, since condylar inclination adjustment affects the cusp height and, to a lesser extent, the occlusal ridge and groove positions [9,10]. Studies have shown that it allows the restoration of the effective shape of the occlusal surface without interferences [2,8]. The steep angles of the articular tubercle allow longer cusps and deeper fossae of the posterior teeth, and shallower concavity of the palatal surfaces of the anterior teeth, thanks to a rapid discussion in the molar area during mandibular movements. A flat articular tubercle requires shorter cusps and shallower grooves of the posterior teeth. The incisal overlap is equally important. The greater the amount of vertical overlap and the smaller the horizontal overlap, the longer the cusps and the deeper the fissures may be [11].

However, there is an ongoing major disagreement between the researchers as the exact relationship between the posterior and anterior determinants has not been clear. Different authors have attempted to confirm or deny the relationship between the posterior determinant—corresponding to the condylar guidance path within the TMJ and the anterior determinant—the incisal, and canine guidance, and their findings vary [12]. The first group claims that there is no correlation between the path of the condyle and the anterior incisal slope [13,14,15]. According to that approach, the anterior guidance must be reconstructed in line with aesthetics, anatomical, and phonetic criteria and provide a posterior disclusion during movements [13]. Dawson emphasizes the importance of adequate concavity or occlusal clearance for anterior guidance to prevent restriction of the mandibular movements and its reduction to simple rotational movement [16].

The opposing group advocates the existence of a connection between the anterior slope and the path of the condyle [12]. As the gnathological approach matured, an anterior guidance steeper than the condylar guidance became a mandatory rule as it provided the elimination of all horizontal forces from the posterior teeth [17]. Brose et al. claim that the incisal guidance angle should always be steeper than the condylar guidance, whereas the height and slope angles of the cusps of the posterior teeth should be harmonious with the condylar and anterior guidance, and where such harmony does not exist, the teeth can be reshaped to a more desirable contour by adjusting or restoring them to eliminate trauma and lessen the harmful effects of parafunction [11]. There are reports suggesting that the steep incisal guidance (IG) may cause the temporomandibular joint to malfunction [18]. It has been hypothesized that IG influences the movements of the condyles, which in turn modifies the growth and morphology of TMJ [19]. However, so far, no one has proven this causation in a meaningful way. Han et al. showed weak but statistically significant correlations between the incisal angle (IGA) and the size of the condyle and fossa centroid [19].

On the other hand, Luca et al. in their study found that there are no differences in the relation between the mandibular fossa features and the inclination of the upper incisors in people with different types of faces, and there is no clinically significant relationship between the shape of the joint and the inclination of the incisors [20]. However, the age of the population in this study ranged from 18 to 40. It is an important factor as TMJ develops between 21 and 23 y.o., and after its peak in growth and development after 17 years of age, TMJ gradually exhibits various modes of adaptation that may be associated with IGA [21]. It is not likely that the morphology and position of incisors have a significant impact on shaping a developing TMJ since incisal guidance seems to be of recent origin. This hypothesis is supported by a study of plague victims in the south of France from 1720 in which a vertical overbite was not present. Some authors argue that incisal guidance did not appear until the Middle Ages, when the use of a fork became common [22,23,24].

### 1.1. Objectives

In view of the controversy raised by the literature, the aim of this study was to investigate the existence of a statistically significant correlation between the incisal features (IGA, interincisal angle, overbite, and overjet), TMJ morphology, and its function.

Therefore, the following null hypotheses were set:

- The protrusive movement of the mandible does correlate with the TMJ anatomy.
- The protrusive movement of the mandible does not correlate with the incisal features.
- The position and relationship of upper and lower permanent incisors do not have a direct and significant effect on the TMJ morphology in young adults.

### 1.2. Clinical Implications

Incisal relationships of permanent dentition should not be considered as impacting TMJ morphology and function at an early age. Therefore, the orthodontist should not overestimate the role of overbite and overjet in the aspect of preventing TMJ disorders, and should focus their attention on more important issues, such as the angle of inclination of the occlusal plane.

If the CBCT scans are available, the AB measurement method allows one to accurately determine the condylar guidance angle and aids in the programming of virtual or analog articulators. Otherwise, electronic axiography is a reliable tool for transferring these data to virtual articulators as a part of prosthetic rehabilitation.

## 2. Materials and Methods

### 2.1. Study Participants

This study was approved by the Independent Bioethics Committee for Scientific Research at Medical University of Gdańsk (number NKBBN/1043/2021-2022) and is retrospectively registered at ClinicalTrials.gov (NCT05637372). Patients received verbal and written information describing the trial and gave their consent to participate in this study. 

Subjects were qualified for this study based on an initial interview including the following criteria: age 21–23 (+/−1), no history of trauma, orthodontic treatment, or TMD. Previous studies by Sulün et al. confirmed that the articular eminence reaches its full size between 21 and 30 years of age in healthy patients and decreases after the age of 31 [25]. Similarly, the articular condyle is fully developed between the ages of 21 and 22 [26]. For this reason, we decided to construct the study group including subjects exactly in 21–23 (+/−1) age range, so the correlation between occlusal and joint features is not falsified by any form of adaptation within the TMJ. The interview was followed by an examination performed in accordance with the Polish version of the RDC/TMD criteria, which disqualified one patient diagnosed with myofascial pain [27]. Eventually, 30 patients qualified for this study.

### 2.2. Study Protocol

For each patient, the angle of the condylar path, incisal guidance angle, the interincisal angle, as well as overbite and overjet were measured on CBCT, followed by the examination with the Modjaw^®^ (MODJAW, Lyon, France) electronic axiograph recording and calculating the functional SCGA for right and left TMJ during the protrusion.

**Figure 1:**
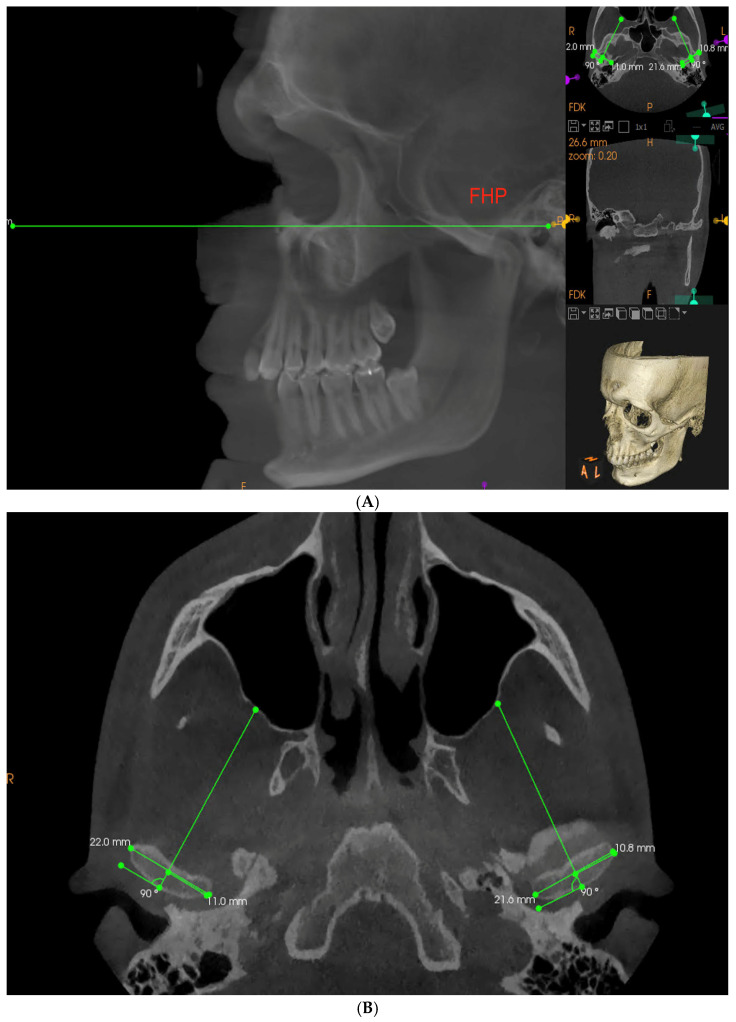
(**A**,**B**) Establishing a Frankfort reference plane and a proper CBCT layer to obtain reproducible measurements for all the patients according to anatomical landmarks (FHP—Frankfort horizontal plane).

Each CBCT examination was conducted by an experienced radiologist technician with the use of Carestream 9300 device (Carestream Dental, Altanta, GA, USA), set to following parameters: 120 kV, 3.20 mA, 40 s with 1698.19 mGy/cm^2^ delivered. The patient was in standing position with the mandible in the maximum intercuspation position (MIP). Obtained images were analyzed in CS Imaging 8.0.5 program. To measure the SCGA on the obtained imaging examination, the Frankfort horizontal plane (FHP) was marked as a horizontal reference plane. It was constructed by connecting the left Orbitale and Porion points on both sides (Figure 1A) [28]. Next, in the sagittal view, a layer perpendicular to one running through the innermost and outermost point of the condyle in transverse cross-section was selected for each joint separately (Figure 1B).

**Figure 2:**
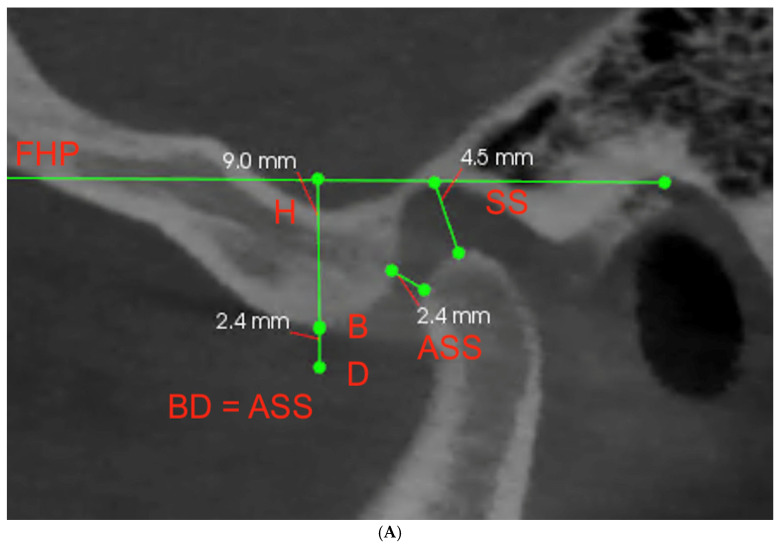

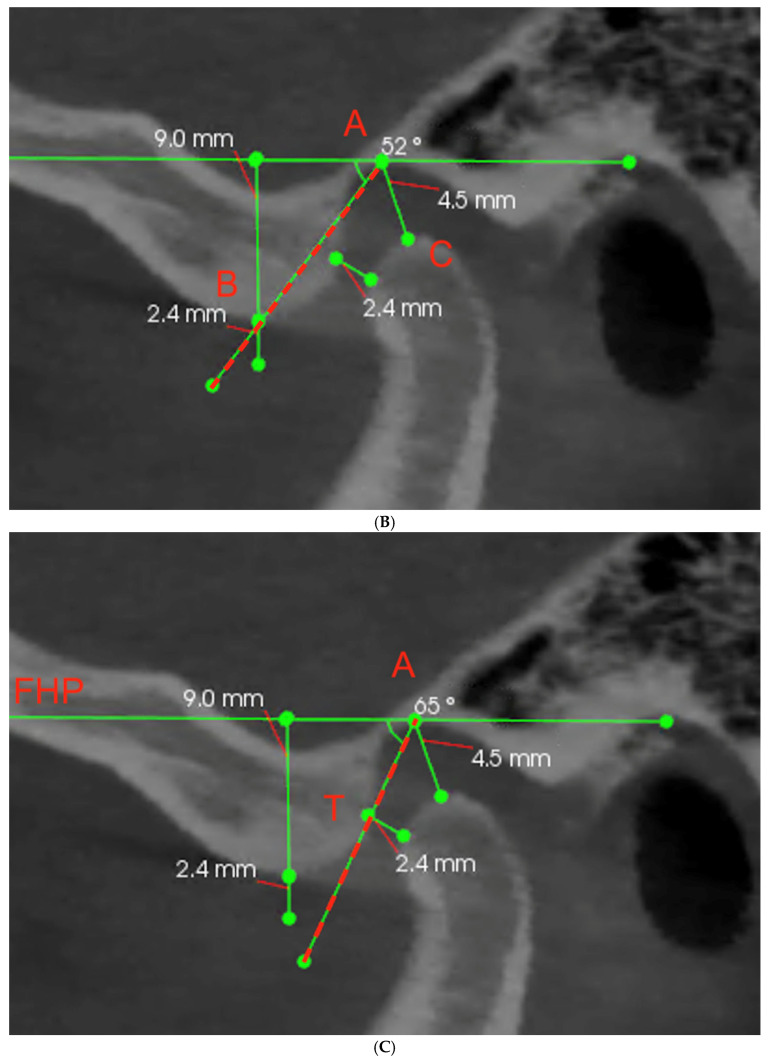

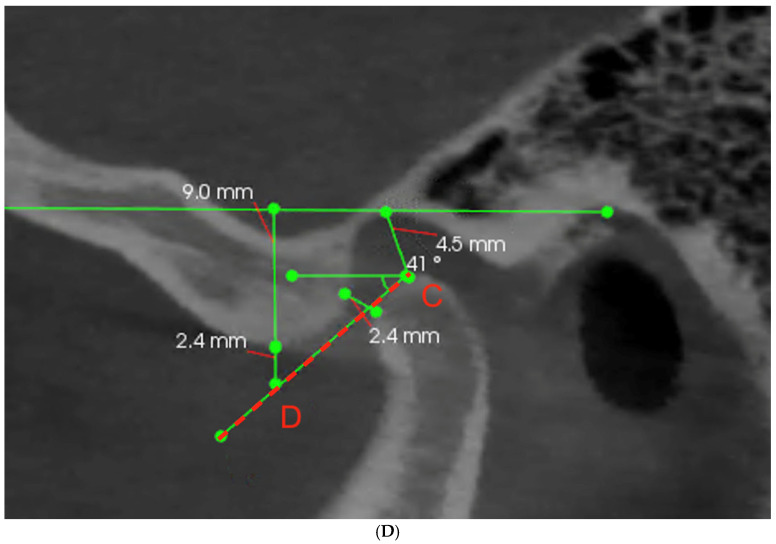
(**A**–**D**) The sagittal view of the TMJ with marked landmarks and illustration of three different methods of measuring the SCGA (ASS—antero-superior space; SS—superior space; H—vertical height of the fossa; FHP—Frankfort horizontal plane; A—deepest point of the articular fossa; B—highest point of the articular eminence; T—tangent point adjacent to the articular eminence; C—highest point of the condyle; D—point below the highest point of the articular tubercle).

The temporomandibular joint space was described by marking the antero-superior space (ASS) and superior space (SS). ASS indicates the thickness of the intermediate band of the disc, while SS indicates the thickness of the posterior band of the disc. Additionally, the vertical height of the fossa (H) was marked (Figure 2A). Then, to achieve the most objective data, three different methods of measuring the SCGA were proposed. First—the AB measurement, which indicates the angle between FHP and the line connecting point A at the deepest point of the articular fossa to point B at the highest point of the articular eminence (Figure 2B). Second—the AT measurement, which indicates the angle between FHP and the line connecting point A at the deepest point of the articular fossa with the tangent point T adjacent to the articular eminence (Figure 2C). Additionally, third—the CD measurement, which indicates angle between Frankfurt plane and a line connecting the highest point C of the condyle to point D, which is below the highest point of the articular tubercle (B), considering the change in the thickness of the disc above the condyle during protrusive movement (Figure 2D). The distance BD reflects intermediate discal space that in vertical dimension is equal to ASS. The numerical data were collected for both joints of each patient and tabulated for further analyses.

**Figure 3:**
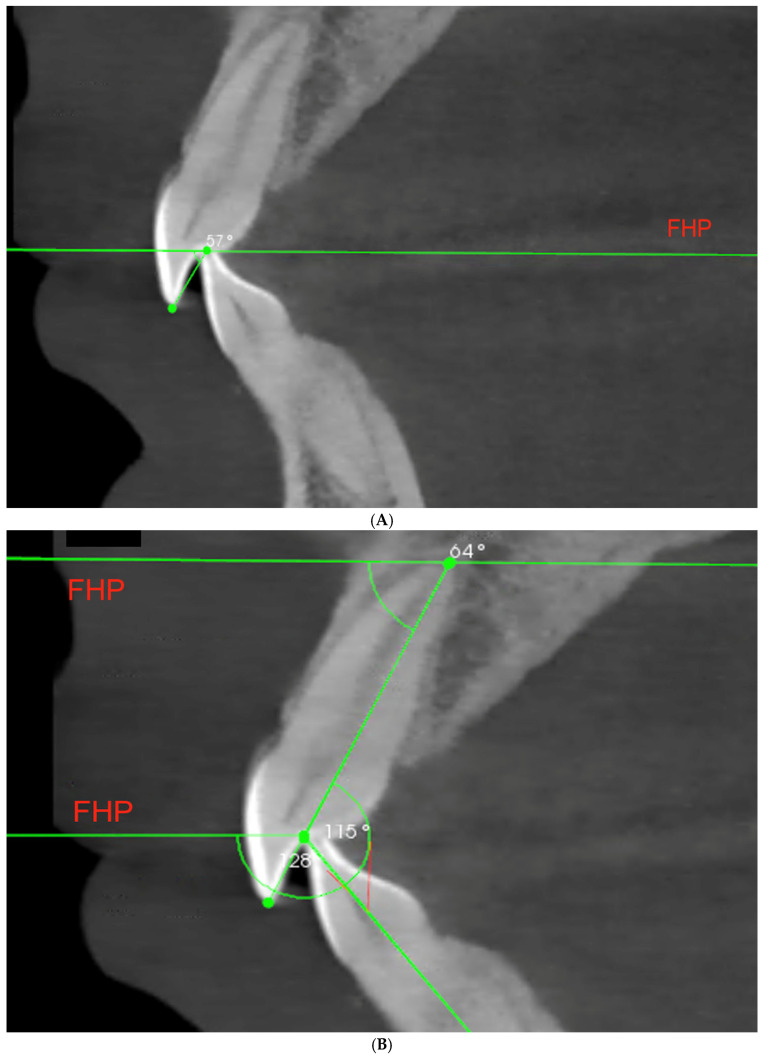

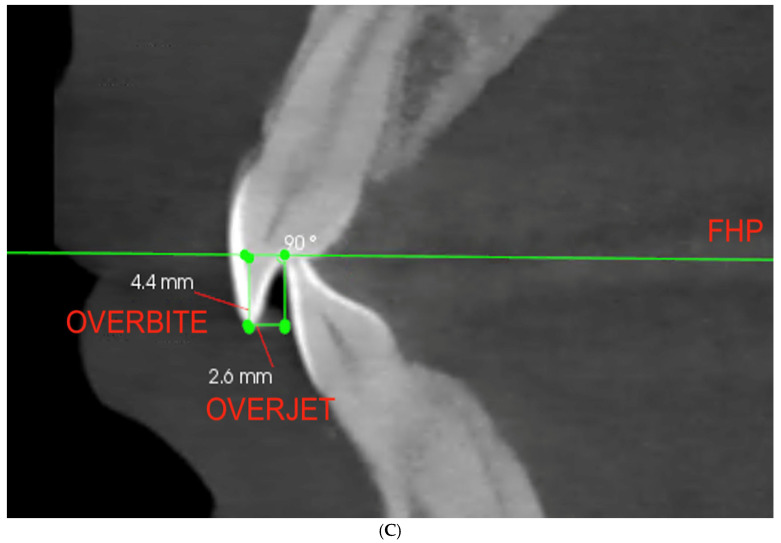
(**A**–**C**) Illustration of measurements concerning the anterior determinant—the incisors: A—incisal guidance angle (IGA), B—interincisal angle, C—overbite and overjet (FHP—Frankfort horizontal plane).

Likewise, the data concerning the incisors were collected. The cross-section view of left and right incisors was examined, and, for each patient, the pair of central incisors presenting the higher value of IGA was selected for further measurements of the incisal features [19,29]. We measured the incisal guidance angle—the angle between the line connecting the incisal margin of the maxillary and mandibular incisors with the FHP (Figure 3A); the interincisal angle—between long axis of upper and lower incisors (Figure 3B); overbite—vertical and perpendicular to FHP distance between incisal margins; and overjet—horizontal and parallel to FHP distance between incisal margin of upper incisor and transverse to the FHP projection of the lower incisal edge (Figure 3C).

**Figure 4:**
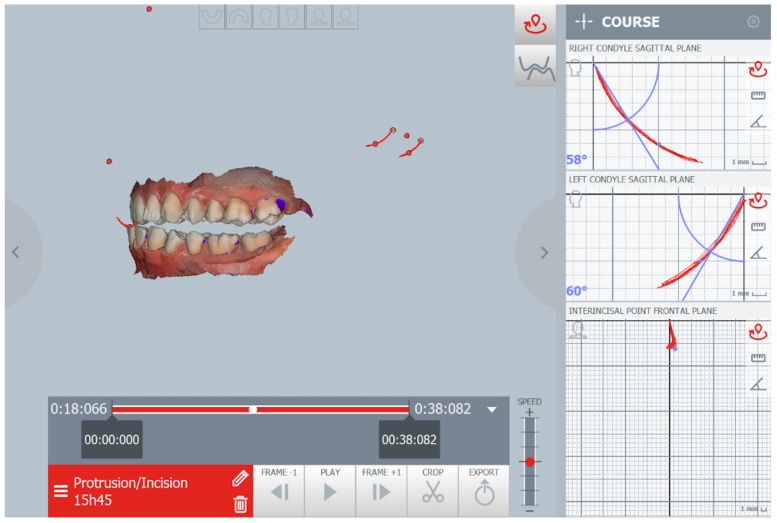
An example of SCGA measured by Modjaw axiography.

Afterward, intraoral scans of qualified patients were conducted with the use of the Carestream 3600 scanner. The obtained STL files were transferred to the Modjaw^®^ measuring device to record the mandible movements in real time (Figure 4). The Modjaw^®^ was designed as a substitute for the facebow, axiograph, and mechanical articulator at the same time, with the possibility to transfer obtained data along with the patient’s individual reference plane to CAD/CAM software (Exocad GmbH, Darmstadt, Germany) [30]. To obtain an individual value of the SCGA, the following actions were performed: fixing the hinge axis by means of repetitive opening and closing movements with the tongue positioned on the palate, and protrusive movement from MIP to edge-to-edge position. The SCGA during function for each patient was computed in the Modjaw^®^ software between the path of the moving condyle and the individual reference plane set by the line connecting the hinge axis to the line created between the Nasion and Subnasale point. 

### 2.3. Statistical Analysis

All calculations have been carried out by means of Microsoft Excel 2019 spreadsheet and STATISTICA (TIBCO Software Inc., Palo Alto, CA, USA; 2020) Data Science Workbench, version 14. In the statistical description of quantitative data, classical measures of location, such as arithmetic means and median, and measures of variation, such as standard deviation and range, were used. The normality of distribution of the variables was tested using Shapiro–Wilk’s test. To assess the linear correlation between two variables, Pearson’s correlation and Spearman’s correlation were used with respect to the type of distribution of the variables tested. To estimate best predictors for a particular dependent variable, multiple regression models were created. In all the calculations, the statistical significance level was set to *p* < 0.05.

### 2.4. Limitations

We are aware of some limitations of this work, which is mostly a small study group, and the fact that the Modjaw^®^ axiograph uses an individual reference pane for each patient. However, due to the heterogeneity of the reference plane and its individual character for each patient, it was not possible to directly compare the angles obtained on Modjaw^®^ and on CBCT; the statistical analysis was applied to check the correlations.

## 3. Results

The measurements of the parameters for all 30 patients (60 TMJ) were collated and subjected to statistical analysis. The aim was to test the correlation between the values of axiographic SCGA during protrusion with SCGA on CBCT (Table 1), the axiographic SCGA during protrusion with the incisal parameters on CBCT (Table 2), and the SCGA with the incisal parameters both measured on CBCT (Table 3), which are presented in the tables below accordingly.

**Figure 5:**
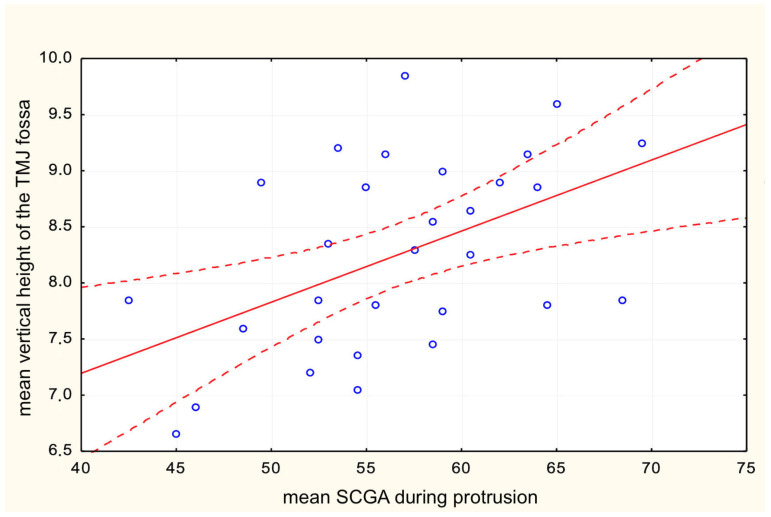
Correlation between mean SCGA during protrusion and mean vertical height of the TMJ fossa.

**Figure 6:**
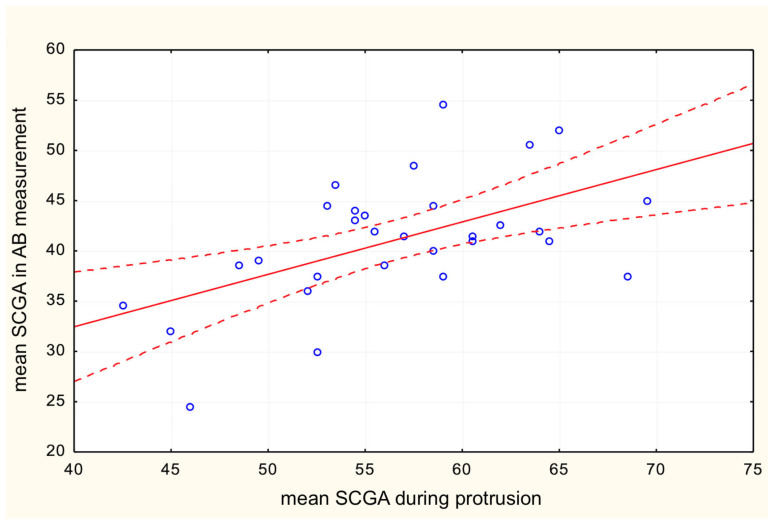
Correlation between mean SCGA during protrusion and mean SCGA in AB measurement.

The results show that the mean functional axiographic measurement of SCGA in protrusion significantly correlates with the TMJ anatomy described by the mean vertical height of the fossa (Figure 5) and the angle of the articular tubercle corresponding to SCGA on CBCT (Figure 6). Moreover, a significant correlation was found between the values of SCGA in the functional and anatomical measurements in all its variants. It turned out that statistically, the AB measurement was the most accurate; however, the AT measurement and the CD method show the least correlation (Table 1). Therefore, this leads to proving the first null hypothesis.

**Figure 7:**
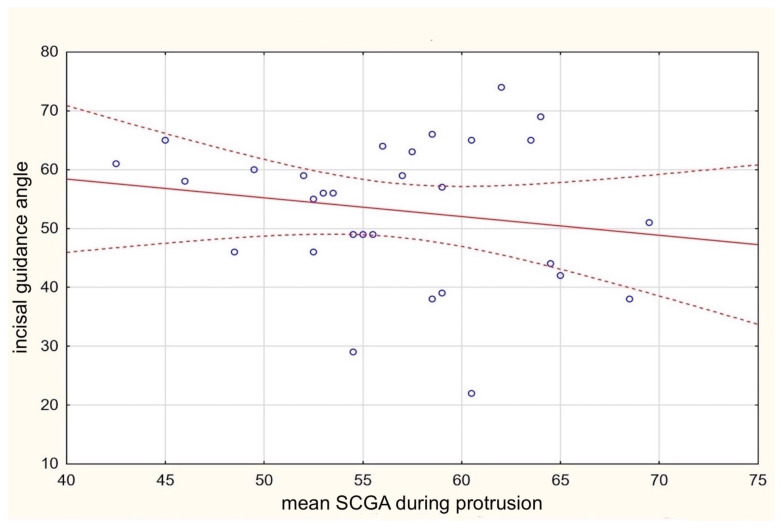
Correlation between mean SCGA during protrusion and the incisal guidance angle.

Subsequent analysis of the mean functional axiographic measurement of SCGA in protrusion collated with the incisal features allowed the confirmation of the second null hypothesis. According to the resolution, the values describing the function of the TMJ did not correlate with any of the incisal parameters: IGA, interincisal angle, overjet, or overbite (Table 2, Figure 7).

**Figure 8:**
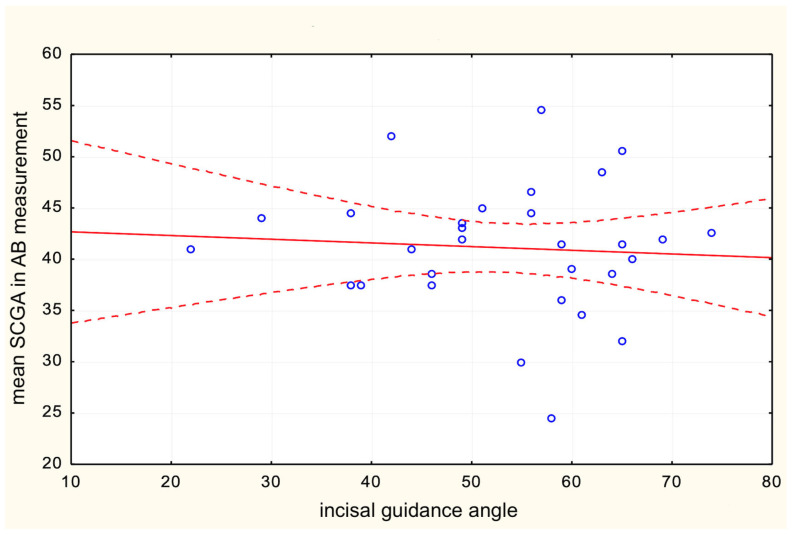
The correlation between the incisal guidance angle and mean SCGA in AB measurement.

Similarly, the third null hypothesis was successfully proved as the incisal parameters did not show a correlation with any of the anatomical CBCT measurements within the TMJ. Regardless of a reference line and slight differences in measurements, no correlation was found between the SCGA on CBCT and front teeth relationships (Table 3, Figure 8).

## 4. Discussion

Many researchers support the assumption that the shape of the temporomandibular joint is related to its function and that specific functional patterns will provide correlating changes in both dentition and TMJ [31,32,33,34]. The lack of a relationship between the position of the incisors and the morphology of the joint, however, is a much more debatable subject. Our results suggest that while the protrusive movement recorded with the axiograph is correlated with the height and inclination angle of the articular tubercle, the position and mutual relations of the front teeth do not show any correlation to the structure of the temporomandibular joint, regardless of which reference points in the joint are used for measurements.

The evident lack of influence of the anterior guidance and overlapping of the anterior permanent teeth on the formation of the temporomandibular joint early in life is not surprising, considering the age range during which the eminence develops and the hypothesis that the overbite appeared only in the Middle Ages. Articular tuberosity develops in more than 50% of people by the age of five, and almost 90% by the age of eight. The remaining 10% of the eminence angle forms during the teenage years [35]. Therefore, it would be surprising if permanent incisors significantly influenced the structure of the TMJ. An interesting example of a relatively recent change was found in the study of the Maïdu Indians from California. This population showed a straight edge-to-edge bite up to colonization. Later studies showed that the occlusion changed to include the incisal bite, “locking” canine occlusion, and these changes happened probably due to the introduction of a soft diet. The study by Laplanche et al., 2010 also showed more than a doubled incidence of class 3 in 1870 and almost doubled incidence of class 2 in 1970 [22].

Another interesting piece of evidence for modern changes in the relationship of the anterior teeth was provided by the linguist Charles Hockett [33]. He suggested that the use of teeth as tools in hunter–gatherer populations used to wear them down; thus, the production of “f” and “v” consonants between the lower lip and upper teeth was much more difficult. Therefore, there was a hypothesis that these sounds were a recent innovation in human language. Blasi et al. confirmed this hypothesis using paleoanthropology, speech sciences, historical linguistics, and methods from evolutionary biology in their research and provided evidence that labiodental sounds (such as “f” and “v”) were introduced after the Neolithic period [36]. It has also been confirmed that the current front teeth overlap is a relatively new phenomenon caused by recent centuries’ dietary modifications. It can therefore be concluded that the soft diet has made a significant contribution to changing the human bite from edge-to-edge configuration to what we now consider to be the norm, including incisal guidance, which some consider mandatory and necessary [37].

Additional evidence of lack of influence of both the bite type and the diet on the TMJ anatomy emerged from a study carried out on 120 human dry skulls divided into four groups: Forest Period Illinois population (900 to 1500 CE); Archaic Period Kentucky population (500 BC to AD 500), African Americans (20th century), and Caucasian Americans (20th century). Both the height and angle of the articular tubercle did not differ significantly between all studied populations. Thus, results do not support the hypothesis of micro-evolutionary changes in the temporomandibular joint caused by changes in the types of food or the way of food preparation over 2500 years [37].

In describing the complicated relationship between the teeth and the temporomandibular joint, one cannot omit the most controversial aspect, which is the impact of occlusion on TMD. Many recent studies report that occlusion is not a significant factor in disorders of masticatory muscles and the TMJ, as it has been repeated many times over the years. Authors in their current research more often highlight the need to evaluate the patients holistically and focus on various factors beyond the head and neck area [38,39,40]. On the other hand, multiple studies present the correlation between occlusion and the prevalence of TMD. A broad view of the etiology and diagnostics of TMD ensures the further development of more effective and modern treatment methods, which underlines a persistent need to reevaluate our knowledge all over in light of more current research [41,42,43,44,45]. Celebic et al. report that different indicators of condylar and incisal guidance affect the activity of anterior and posterior temporal muscles [30]. Tinastepe et al. found that patients with increased vertical overlapping occlusions and minimal horizontal overlap had more clinical symptoms associated with TMD than patients with physiological mandibular morphology [46]. In a study by Williamson et al., it was reported that masseter and temporalis muscle activity could only be reduced when anterior guidance causes disclusion in the molar region [47]. In the same study, authors stated that muscle activity is not eliminated by the specific relationships of the anterior teeth but by the elimination of contacts on the molars, which is also true for the therapy with the use of anterior jigs [48,49]. Obtaining an immediate disclusion in the posterior region with a protrusive movement may also protect the structure of the posterior teeth by the elimination of the balancing contacts; thus, incisal guidance is still considered to be an important factor in prosthetic rehabilitation and reconstruction. However, it still is a controversial subject whether the existence of balancing contacts can be a cause of TMD. Interestingly, a study by Kahn et al. proves that the balancing occlusal contacts were present much more often in patients without TMD, whereas the patients presenting the symptoms associated with TMD would mostly show an uninterrupted canine guidance [50]. A possible explanation of such an outcome could be the fact that people with TMD tend to clench more, whereas healthy, asymptomatic people grind more often, so they are more likely to show signs of interferences and group contacts during eccentric movements [51]. Moreover, it was proven in a 30 years long cohort study including 1037 patients that abnormal occlusal features such as posterior crossbite and high and low overbite in adolescence are not associated with a higher incidence of TMD later in life [52].

Additionally, according to the results of previous longitudinal studies, high overbite (≥4 mm) at the age of 15 was negatively associated with TMJ abnormalities at the age of 45 [53]. It is also worth answering the question of whether deep bite really protects the TMJ. It turns out that a high overbite is a feature common among people with hypodivergent facial patterns, who generally have larger temporomandibular joint condyles than hyperdivergent individuals [54]. There is a hypothesis that large condyles, due to the larger force distribution area, are less susceptible to mechanical stress than small ones, thus protecting against subsequent disc disturbances [55]. This hypothesis seems to be consistent with the results of a systematic review by Manfredini et al., indicating that facial hyperdivergence is a risk factor for degenerative disorders and temporomandibular disc dysfunction [56]. The following points toward the conclusion that the relationship of the incisors in terms of TMD might be significantly less important than the initial cause of their disrupted relationship, which is the vertical growth pattern. This pathology is also associated with a less favorable composition of the muscle fibers. The fibrous composition of the masseter muscle was studied based on a biopsy performed during the surgical correction of malocclusion. It turned out that the occupancy of type I fibers and areas of hybrid I/II fibers increased in open bites, and vice versa; the occupancy of type II fibers increased in deep bites [57]. This means that the masseter muscles of people presenting with an open bite are not predisposed to long and firm contractions characteristic of clenching activity. Further confirmation that skeletal abnormalities, rather than anterior tooth relationships, may contribute to TMD emerged in a study by Cifter et al. in 2022 [58]. It was found that clockwise jaw rotation, a frequent consequence of disrupted vertical facial growth, causes increased compression within the TMJ. Focusing on the occlusion, it could be once again considered that an impaired anterior teeth relationship is the cause of the muscular disorders, while it appears to be only secondary to skeletal disorders caused by abnormal facial growth.

## 5. Conclusions

1. The SCGA, during the protrusive movement recorded by the axiograph, is correlated with the features of the articular fossa, such as the height and inclination angle of the articular tubercle, suggesting that the TMJ anatomy dictates its function.
2. The SCGA, during the protrusive movement, is not correlated with the relations between the incisors, pointing towards the conclusion that the TMJ function does not depend on the incisal features (IGA, interincisal angle, overbite, and overjet).
3. Incisal relationships of permanent teeth such as overbite, overjet, IGA, and interincisal angle do not correlate with TMJ anatomy; therefore, they do not affect TMJ formation in young adults in regard to analyzed study group.
4. The AB line is the most reliable reference for measuring the SCGA on CBCT.
5. While processed foods contributed significantly to the change in the bite, neither a soft diet nor a change in the bite modified the structure of the temporomandibular joint, either now or in antiquity.

## Figures and Tables

**Table 1:** Correlation between mean SCGA during protrusion and mean SCGA in CBCT measurements.

Variables	*n*	Mean	SD	r (X, Y)	r^2^	*p*
Mean SCGA during protrusion	30	56.6000	6.6299	0.496253	0.246267	0.005285
Mean vertical height of the TMJ fossa		8.2467	0.8467			
Mean SCGA during protrusion	30	56.6000	6.6299	0.551815	0.304500	0.001571
Mean SCGA in AB measurement		41.1167	6.2694			
Mean SCGA during protrusion	30	56.6000	6.6299	0.532373	0.283421	0.002459
Mean SCGA in AT measurement		52.4167	7.9525			
Mean SCGA during protrusion	30	56.6000	6.6299	0.449854	0.202368	0.012623
Mean SCGA in CD measurement		36.4833	5.8509			

**Table 2:** Correlation between mean SCGA during protrusion and the incisal parameters measured on CBCT.

Variables	*n*	Mean	SD	r (X, Y)	r^2^	*p*
Mean SCGA during protrusion	30	56.6000	6.6299	−0.173606	0.030139	0.358898
Overbite		3.5400	1.2544			
Mean SCGA during protrusion	30	56.6000	6.6299	0.022152	0.000491	0.907500
Overjet		2.4067	0.7701			
Mean SCGA during protrusion	30	56.6000	6.6299	−0.183319	0.033606	0.332212
Interincisal angle		134.6000	11.7403			
Mean SCGA during protrusion	30	56.6000	6.6299	−0.173343	0.030048	0.359636
Incisal guidance angle		53.1333	12.1789			

**Table 3:** The correlation between the incisal guidance angle and mean SCGA in AB measurements.

Variables	*n*	Mean	SD	r (X, Y)	r^2^	*p*
Incisal guidance angle	30	53.13333	12.17893	−0.069985	0.004898	0.713254
Mean SCGA in AB measurement		41.11667	6.26936			
Incisal guidance angle	30	53.13333	12.17893	−0.062899	0.003956	0.741250
Mean SCGA in AT measurement		52.41667	7.95254			
Incisal guidance angle	30	53.13333	12.17893	−0.129900	0.016874	0.493869
Mean SCGA in CD measurement		36.48333	5.85085			

## Data Availability

Not applicable.

## Abbreviations

*Abbreviation*	*Meaning*
TMJ	Temporomandibular joint
TMD	Temporomandibular disorders
IGA	Incisal guidance angle
CBCT	Cone beam computed tomography
SCGA	Sagittal condylar guidance angle
SCG	Sagittal condylar guidance
IG	Incisal guidance
MIP	Maximum intercuspation position
FHP	Frankfort horizontal plane
ASS	Antero-superior space
SS	Superior space
